# Identification and Structural Elucidation of a Novel Pyrrolidinophenone-Based Designer Drug on the Illicit Market: α-BPVP

**DOI:** 10.1021/acs.chemrestox.5c00068

**Published:** 2025-05-07

**Authors:** Sara Casati, Roberta F. Bergamaschi, Riccardo Primavera, Alessandro Ravelli, Ivana Lavota, Alessio Battistini, Gabriella Roda, Chiara Ciccarelli, Claudio Guidotti, Paola Rota

**Affiliations:** † Dipartimento di Scienze Biomediche, Chirurgiche ed Odontoiatriche, Università degli Studi di Milano, 20133 Milan, Italy; # Fondazione IRCCS Ca’ Granda Ospedale Maggiore Policlinico, 20122 Milan, Italy; $ Dipartimento di Scienze Biomediche per la Salute, Università degli Studi di Milano, 20133 Milan, Italy; ° Institute for Molecular and Translational Cardiology (IMTC), San Donato Milanese, 20097 Milan, Italy; £ Gabinetto Regionale di Polizia Scientifica per la Lombardia, Polizia di Stato, Via Fatebenefratelli 11, 20121 Milan, Italy; ^ Dipartimento di Scienze Farmaceutiche, Università degli Studi di Milano, 20133 Milan, Italy

## Abstract

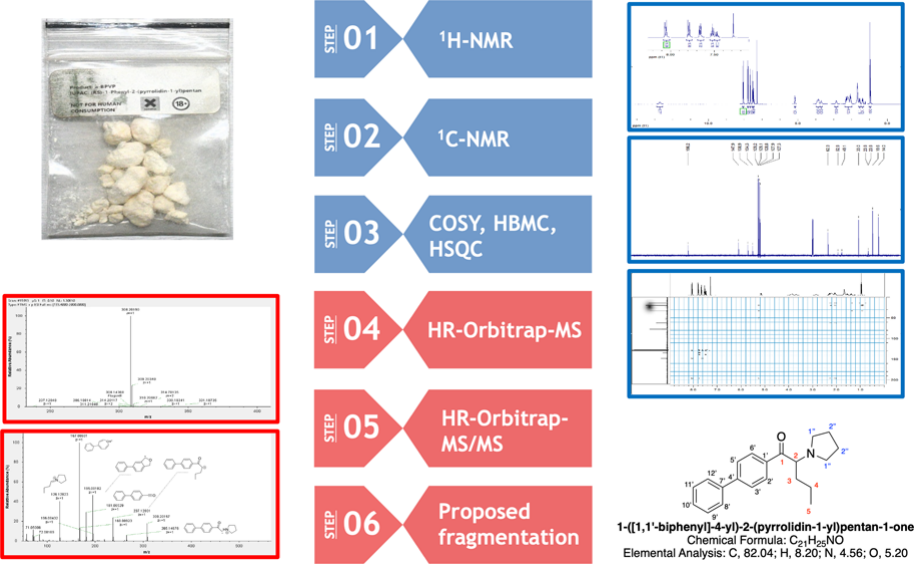

The identification of a new psychoactive substances (NPS) with a cathinone structure and a biphenyl substituent, found in seized powder from the black market, is here reported. By combining analytical techniques, including 1D and 2D NMR and HRMS, the compound was identified as 1-([1,1′-biphenyl]-4-yl)-2-(pyrrolidin-1-yl)pentan-1-one (α-BPVP), an α-pyrrolidinopentiophenone (α-PVP) analogue featuring a biphenyl group instead of the phenyl ring. This previously unreported molecule raises urgent legal and public health concerns, which warrants further toxicological investigation.

The new psychoactive substances (NPS) phenomenon represents a pivotal challenge compared to traditional drugs, emerging as a global threat to public health and institutions.^1^ Synthetic cathinones and cannabinoids as well as phenethylamines and piperazines are some examples of NPS classes. The health risks of novel compounds are largely unknown, with some posing serious or fatal risks to consumers. Few studies about NPS toxicity and their correlation between their consumption and psychopathological symptoms have been conducted;^2−4^ however, research on these substances is challenging, due to their continuous change and the lack of analytical certified materials. Other difficult aspects of the NPS phenomenon regard the recognition from law enforcement during a seizure and the legislation: NPS are not controlled by international legislative control conventions; however, in Europe and non-EU countries, some classes of NPS may be subject to national regulatory measures.^5^ For this reason, the market for NPS is constantly expanding since each year new substances are created to avoid legal controls. Even though this phenomenon is slow, reports of already encountered NPS are increasing. Common approaches to identify NPS are gas chromatography (GC) and liquid chromatography (LC) separation coupled to mass spectrometry (MS) detection. Moreover, considering the rapid evolution of NPS, the use of updated libraries is fundamental for the recognition. However, when new molecules are dropped on the market, the combined use of different techniques may be required to gather complementary data supporting the submitted case. In our case, a 38-year-old man was found dead in his room without any signs of injuries and trauma but with some paraphernalia and wrappers containing powders around him. The police seized all and multiple already known NPS (3,4-MDPHP, 3,4-MDiPHP, α-PCYP and 2-MMC) were identified. However, the fragmentation of a white powder included in a packet with a tag reporting the name “a-BPVP” did not match any compounds in the GC–MS libraries. In this case, the elucidation of unknown substances requires the combined use of high-resolution mass spectrometry (HRMS) and nuclear magnetic resonance (NMR). Indeed, HRMS data alone may be insufficient to provide detailed structural information, necessitating the use of two-dimensional NMR experiments.

In this paper, we describe the identification and characterization of an α-PVP analogue (Figure 1), the α-BPVP in a seized powder by different analytical techniques, i.e., GC–MS with electron impact ionization (GC-EI/MS), high-resolution accurate-mass Orbitrap mass spectrometry (HRAM-Orbitrap-MS), and 1D and 2D NMR analyses. Remarkably, we report the first identification of a previously unknown NPS: 1-([1,1′-biphenyl]-4-yl)-2-(pyrrolidin-1-yl)pentan-1-one. This discovery raises significant legal and health concerns, highlighting the urgent need for monitoring and further investigation of its toxicological and public health impact.

**Figure 1:**
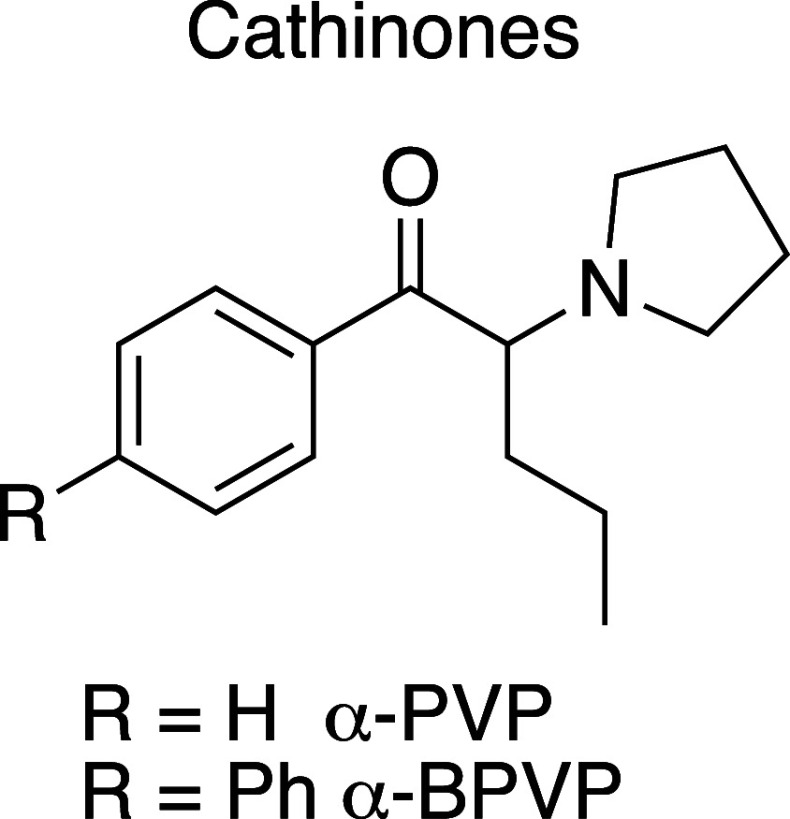
Structure of synthetic cathinones: α-PVP and the new derivatives α-BPVP.

The seized powder, labeled “a-BVPV” on the packaging as found on the market for psychoactive substances, was initially analyzed using routine analytical techniques, including GC–MS and quadrupole mass spectrometry, to identify the molecule via database library matching. The inability to achieve conclusive identification with these methods necessitated a comprehensive NMR analysis for structural elucidation.

^1^H NMR analysis was performed in both CDCl_3_ and CDCl_3_ with a 1:1 methanol mixture. Pure chloroform allowed the observation of the NH proton at downfield regions (12.73 ppm), confirming that the powder was in salt form; on the other hand, the latter solvent system improved compound solubility and was initially selected for the complete 1D and 2D characterization.

The ^1^H NMR analysis immediately indicated a cathinonic structure (Figure 2), showing a triplet associated with the hydrogen at the α-carbon of the carbonyl group at 5.47 ppm (H at C2), an aromatic system, signals corresponding to an alkyl chain, and peaks consistent with a pyrrolidine ring (between 3.71 and 3.18 and 2.22–2.06 ppm). Notably, by arbitrarily assigning an integration value of one to the hydrogen at 5.47 ppm, the analysis confirmed the presence of a pyrrolidine ring (4H at C1″/C4″ and 4H at C2″/C3″) and a three-carbon alkyl chain, while simultaneously revealing an unexpected nine aromatic hydrogen atoms between 8.16 and 7.36 ppm (see the Supporting Information).

**Figure 2:**
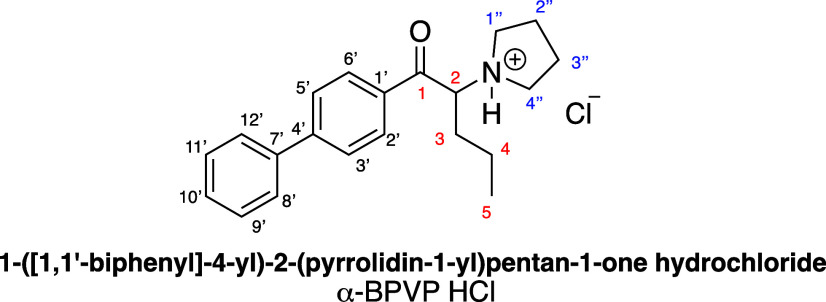
Numbering of the α-BPVP HCl salt structure.

Moreover, the ^13^C NMR analysis revealed the presence of the carbonyl function at 195.9 ppm and the α-carbon to the carbonyl group at 67.7 ppm. It also confirmed the existence of an extended aromatic system (139.4–127.5 ppm), an alkyl chain presumably composed of three carbon atoms (at 33.0, 18.3, and 14.0 ppm), and the pyrrolidine ring (2C at lower field 54.1, 52.6 and 2C at higher ones at 23.6 and 23.5 ppm). This suggested a structure similar to that of α-PVP, but differing in the aromatic portion.

Therefore, bidimensional NMR experiments were essential to accurately assign the structure, better understand the linkage between the aromatic rings, and verify the other substituents. In particular, the assignments relied on chemical shifts (δ, ppm) for ^1^H and ^13^C, proton resonance multiplicity patterns determined by *J* couplings (Hz), and correlations observed in the ^1^H–^1^H (COSY) and ^1^H–^13^C (HSQC) spectra. Based on the couplings observed in the COSY and in the HSQC, the propyl chain was confirmed, together with the presence of a pyrrolidine ring. Notably, the COSY spectrum indicated an aromatic AA′BB′ system consistent with a para-substituted aromatic ring alongside a second unsubstituted aromatic ring.

Moreover, to determine whether the two aromatic rings were connected, long-range ^1^H–^13^C correlations were investigated through HMBC experiments. The carbonyl group at C1 couples with the para-substituted aromatic ring, as clearly indicated by the coupling between the carbonyl carbon and H-2′ and H-6′ (195.9 and 8.13 ppm). Moreover, C4′ shows a strong coupling with both H-2′ and H-6′ (148.2 and 8.13 ppm) as well as with H-8′ and H-12′ (148.2 and 7.61 ppm). Similarly, C7′ couples with H-8′ and H-12′ (139.4 and 7.61 ppm) and with H-3′ and H-5′ (139.4 and 7.76 ppm), demonstrating the linkage between the aromatic rings to form a biphenyl system.

At this stage, an accurate mass analysis became necessary to confirm the molecular mass of the compound. The analysis was performed using a QExactive Plus Orbitrap MS (Thermo Fisher Scientific) equipped with an Ion Max source (Thermo Fisher Scientific). The direct infusion of the unknown sample in positive full scan mode revealed an *m*/*z* ratio of 308.2021, corresponding to [M + H]^+^. This is consistent with the elemental composition of α-BPVP (C_21_H_26_NO^+^) and a calculated exact mass of 308.2009.

Considering that NPS follow similar fragmentation pathways as reported for synthetic cathinones,^6,7^ MS/MS experiments were performed at different collision energies (25, 35, and 50 eV), confirming that the unknown compound was a synthetic cathinone. Moreover, based on this fragmentation analogy, it was hypothesized that the molecule fragments were similar to those reported for the α-PVP (see the Supporting Information).

Specifically, a fragment with a mass of 237.1274 is observed, likely due to the loss of the pyrrolidine ring (−NC_4_H_9_). The subsequent loss of the alkyl chain generates an ion with a mass-to-charge ratio (*m*/*z*) of 195.0804. This ion can further rearrange into a substituted tropylium cation with an *m*/*z* of 167.0855, representing the fragment with the highest intensity. Other significant fragments included ions with *m*/*z* values of 126.1277 and 84.0808, both attributable to the portion containing the pyrrolidine ring. This fragmentation pattern was consistent with that previously observed for the fragmentation of α-PVP.^7^ However, the proposed fragmentation pattern remains highly speculative, and further investigations using isotopically labeled molecules would be necessary.

Finally, for a complete characterization, the molecule was also evaluated for optical rotation as well as its melting point. The low observed optical rotation, close to zero, suggests a mixture of the two enantiomeric forms, R and S, as indicated on the seized package with a slight enantiomeric excess of one of the isomers. However, further HPLC studies using chiral columns are necessary to confirm this observation. Moreover, the high melting point, ranging between 223 and 228 °C, confirms that the molecule is in its salt form. To determine the nature of the counterion, preliminary qualitative assays for halides were performed, which indicated the presence of chloride ions. The fact that the compound is in its hydrochloride form was further confirmed by ^35^Cl NMR analysis, performed at 49 MHz, which clearly detected the chloride nucleus (see the Supporting Information).

In conclusion, this represents the identification and complete characterization of a new substance sold on the illegal market as a drug with a cathinone-like structure. Further studies on the pharmacological activity of this molecule and its toxic effects are ongoing.

## Experimental Section

No unexpected or unusually high safety hazards were encountered.

### Chemicals

All chemicals and solvents used were of analytical grade and purchased from Sigma-Aldrich (St. Louis, MO, USA). The ^1^H NMR data are tabulated in the following order: multiplicity (s = singlet, d = doublet, t = triplet, br = broad, m = multiplet, app = apparent), coupling constant(s) given in hertz, number of protons, and assignment of proton(s).

### Selected Information α-BPVP

Mp 223–228 °C; [α]_D_^23^ = −7.5 (*c* = 1.0 in methanol); ^1^H NMR (500 MHz, CDCl_3_/CD_3_OD 1:1 v/v) δ = 8.13 (d, *J* = 8.5 Hz, 2H; H-2′ and H-6′), 7.76 (d, *J* = 8.5 Hz, 2H; H-3′ and H-5′), 7.61 (d, *J* = 7.1 Hz, 2H; H-8′ and H-12′), 7.45 (t app, *J* = 7.1 Hz, 2H; H-11′ and H-9′), 7.41–7.36 (m,1H; H-10′), 5.78 (t app, *J* = 6.0 Hz, 1H; H-2), 3.71–3.60 (overlapping, 2H; H-1″ or H-4″), 3.38–3.18 (overlapping, 2H; H-1″ or H-4″), 2.22–2.06 (overlapping, 4H; H-2″ and H-3″), 2.06–1.96 (overlapping, 2H; H-3a and H-3b), 1.37–1.21 (overlapping, 2H; H-4a and H-4b), 0.85 ppm (t, *J* = 7.3 Hz, 3H; H-5); ^13^C NMR (125 MHz, CDCl_3_/CD_3_OD 1:1 v/v) δ = 195.9 (C1), 148.2 (C4′), 139.4 (C7′), 133.4 (C1′), 129.9 (2C, C-6′ and C-2′), 129.4 (2C, C-11′ and C-9′), 129.1 (C10′), 128.2 (2C, C-5′ and C-3′), 127.5 (2C, C-12′ and C-8′), 67.7 (C2); 54.1 and 52.6 (2C, C1″ and C4″), 33.0 (C3), 23.6 and 23.5 (2C, C2″ and C3″), 18.3 (C4), 14.0 ppm (C5); HRMS (HESI/Orbitrap), *m*/*z*, [M + H]^+^ Calcd for C_21_H_26_NO^+^ 308.2009, Found 308.2021. Complete information can be found in the Supporting Information.

## Supporting Information Available

Material and methods, NMR and, HRMS, GC–MS and HPLC–MS spectra (PDF)

## Abbreviations

NPS: new psychoactive substances
α-PVP: α-pyrrolidinopentiophenone
α-BPVP: 1-([1,1′-biphenyl]-4-yl)-2-(pyrrolidin-1-yl)pentan-1-one
NMR: Nuclear Magnetic Resonance
HRMS: High-Resolution Mass Spectrometry
GC: Gas Chromatography
LC: Liquid Chromatography
3,4-MDPHP: 3,4-methylenedioxy-α-pyrrolidinohexanophenone
3,4-MDPiHP: 3,4-methylenedioxy-α-pyrrolidinoisohexanophenone
2-MMC: 2-methylmethcathinon
α-PCYP: α-pyrrolidinocyclohexanophenone
COSY: COrrelation SpectroscopY
HSQC: Heteronuclear Single Quantum Coherence
HMBC: Heteronuclear Multiple Bond Correlation
