# Supplementary material for: Identification and Structural Elucidation of a Novel Pyrrolidinophenone-Based Designer Drug on the Illicit Market: α-BPVP
Source: Chem Res Toxicol. 2025 May 7;38(5):808–11. doi: 10.1021/acs.chemrestox.5c00068 (PMC12093358; doi:10.1021/acs.chemrestox.5c00068)
Supplement: Supplementary file 1 — tx5c00068_si_001.pdf [file tx5c00068_si_001.pdf]

Supporting Information

Identification and Structural Elucidation of A Novel Pyrrolidinophenone-Based Designer Drug on the illicit market: α-BPVP.

Sara Casati[†,#], Roberta F. Bergamaschi[†], Riccardo Primavera[$], Alessandro Ravelli[†], Ivana Lavota[°], Alessio Battistini[†], Gabriella Roda[^], Chiara Ciccarelli[£], Claudio Guidotti[£], Paola Rota[*,†,°]

† Dipartimento di Scienze Biomediche, Chirurgiche ed Odontoiatriche, Università degli Studi di Milano, 20133 Milan, Italy

Fondazione IRCCS Ca' Granda Ospedale Maggiore Policlinico, 20122, Milan, Italy

$ Dipartimento di Scienze Biomediche per la Salute, Università degli Studi di Milano, 20133, Italy

° Institute for Molecular and Translational Cardiology (IMTC), San Donato Milanese, Milan, 20097, Italy

£ Gabinetto Regionale di Polizia Scientifica per la Lombardia, Polizia di Stato, Via Fatebenefratelli 11, 20121 Milan, Italy

^ Dipartimento di Scienze Farmaceutiche, Università degli Studi di Milano, 20133, Italy

Corresponding author e-mail: *paola.rota@unimi.it

**Contents**	**Page Number**
Material and methods	S2-S3
NMR tabulation	S4-S10
^{1}H-NMR in $CDCl_3$:MeOD, 1:1, v/v	S4
^{1}H-NMR in $CDCl_3$	S5
^{1}H-NMR in $CDCl_3$:MeOD, 1:1, v/v	S6
^{13}C-NMR in $CDCl_3$	S7
COSY in $CDCl_3$:MeOD, 1:1, v/v	S8
HSQC in $CDCl_3$	S9
HMBC in $CDCl_3$	S10
^{35}Cl-NMR in CD_3OD:D_2O	S11
HRMS full scan spectra	S12
HRMS-MS spectra and fragments attribution	S13-14
GC-MS spectra	S15

Material and methods

Chemicals. All chemicals and solvents used were of analytical grade and purchased from Sigma-Aldrich (St. Louis, MO, USA).

NMR:

Nuclear magnetic resonance spectra were recorded at 298 K on a Bruker AM-500 spectrometer equipped with a 5 mm inverse-geometry broadband probe and operating at 500.13 MHz for ^{1}H, 125.76 MHz for ^{13}C and 49.00 MHz for ^{35}Cl. Chemical shifts are reported in parts per million and are referenced for ^{1}H spectra to a solvent residue proton signal (δ = 3.31 ppm for CD_3OD or δ = 7.26 ppm for $CDCl_3$) and for ^{13}C spectra, to solvent carbon signal (central line at 49.05 ppm, for CD_3OD or δ = 77.0 ppm for $CDCl_3$). ^{35}Cl NMR chemical shifts were referenced using an external tube containing aqueous HCl in CD_3OD as the reference standard, to which an arbitrary value of 0 ppm was assigned. Proton and carbon assignments were established, with ^{1}H-^{1}H and ^{1}H-^{13}C correlated NMR experiments. ^{1}H NMR data are tabulated in the following order: number of protons, multiplicity (s, singlet; d, doublet; t, triplet, t app, apparent triplet, br s, broad singlet; m, multiplet), coupling constant(s) in hertz, assignment of proton(s).

The numbering was done according to the figure:

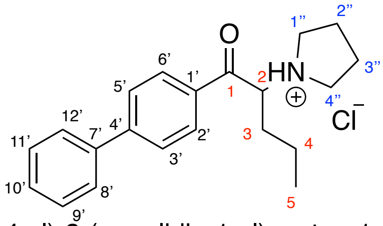

1-([1,1'-biphenyl]-4-yl)-2-(pyrrolidin-1-yl)pentan-1-one hydrochloride

HRMS:

QExactive Plus Orbitrap MS (Thermo Fisher Scientific) equipped by an Ion Max source (Thermo Fisher Scientific) was used to analyze the compound. The HESI spray voltage was set to 3.70 kV. The ion transfer capillary was heated to 320 °C to desolvate droplets. The S-lens RF level was set at 50. The sheath gas flow rate 25, auxiliary gas flow rate 10, sweep gas flow rate 0, auxiliary gas heater temperature 310 °C. For the full scan, settings were: resolution of 70,000, 50 ms maximum injection time, Automatic Gain Control (AGC) target 1 × 106, scan range 50.0-755.0 m/z, positive mode. The MS^2 of precursor ion settings were: resolution of 35.00, AGC target of 1×10^6. The precursor ions fragmentation if it had an unassigned charge state or the charge states +1.

Optical rotation:

The optical rotation was taken on a PerkinElmer 241 polarimeter equipped with a 1 dm tube and the $[\alpha]_D$ values are given in 10^{-1} deg cm^2/g and the concentrations are given in g per 100 mL.

GC-MS:

GC-MS analysis was performed on an Agilent 6890N gas chromatograph interfaced with a single quadrupole 5975N detector (Palo Alto, CA, USA). The GC separation was carried out on an Agilent capillary column Varian CP-Sil8 (15 m length × 0.25 mm i.d., 0.25 μm film thickness) using the following oven temperature program: from 70 °C (held 2 min) to 160° at 40 °C/min, then to 290 °C at 8°C/min (held 2.50 min). The injector temperature was 270 °C, the ion source temperature was 300

°C; carrier gas (helium) flow was 1.1 mL/min; the injection mode was splitless; the injection volume was 2 μL; the run time was 23.00 min, and the mass spectrometer mode was electron ionization conditions (70 eV) by full scan mode (range 40 – 600 m/z).

α-BPVP NMR

^{1}H-NMR (500 MHz, $CDCl_3/CD_3OD$ 1:1 v/v) δ = 8.13 (2H, d, J = 8.5 Hz; H-2' and H-6'), 7.76 (2H, d, J = 8.5 Hz; H-3' and H-5'), 7.61 (2H, d, J = 7.1 Hz; H-8' and H-12'), 7.45 (2H, t app, J = 7.1 Hz; H-11' and 9'), 7.41-7.36 (1H, m; H-10'), 5.78 (1H, t app, J = 6.0 Hz; H-2), 3.71-3.60 (2H, overlapping, H-1"a and H-4"a) 3.38-3.18 (2H, overlapping, H-1"b and H-4"b), 2.22-2.06 (4H, overlapping; H-2"and H-3"), 2.06-1.96 (2H, overlapping; H-3a and H-3b), 1.37-1.21 (2H, overlapping; H-4a and H-4b), 0.85 (3H, t, J = 7.3 Hz; H-5); ^{13}C-NMR (125 MHz, $CDCl_3/CD_3OD$ 1:1 v/v) δ = 195.9 (C1), 148.2 (C4'), 139.4 (C7'), 133.4 (C1'), 129.9 (2C, C-6' and C-2'), 129.4 (2C, C-11' and C-9'), 129.1 (C10'), 128.2 (2C, C-5' and C-3'), 127,5 (2C, C-12' and C-8'), 67.7 (C2); 54.1 and 52.6 (2C, C1" and C4"), 33.0 (C3), 23.6 and 23.5 (2C, C2"and C3"), 18.3 (C4), 14.0 (C5).

^{1}H-NMR (500 MHz, $CDCl_3$) δ = 12.73 (1H, s; NH), 8.06 (2H, d, J = 8.5 Hz; H-2' and H-6'), 7.79 (2H, d, J = 8.5 Hz; H-3' and H-5'), 7.66 (2H, d, J = 7.1 Hz; H-8' and H-12'), 7.52 (2H, t app, J = 7.1 Hz; H-11' and 9'), 7.47 (1H, m; H-10'), 5.16 (1H, m; H-2), 3.97-3.75 (2H, overlapping, H-1"a and H-4"a) 3.68 (1H, br s, H-1"b or H-4"b), 2.84 (1H, br s, H-1"b or H-4"b), 2.37-1.97 (6H, overlapping H-2"a,b,c,d and H-3a and H-3b), 1.54 (1H, m, H-4a), 1.37 (1H, m, H-4b), 0.95 (3H, t, J = 7.3 Hz; H-5); ^{13}C-NMR ($CDCl_3/CD_3OD$ 1:1 v/v) δ = 196.2 (C1), 147.9 (C4'), 138.9 (C7'), 134.3 (C1'), 129.2 (2C, C-6' and C-2'), 129.1 (2C, C-11' and C-9'), 128.8 (C10'), 127.9 (2C, C-5' and C-3'), 127.3 (2C, C-12' and C-8'), 62.3 (C2), 52.9 and 49.1 (2C, C1" and C4"), 33.0 (C3), 23.9 (2C, C2"and C3"), 19.6 (C4), 14.0 (C5).

Optical rotation

$[\alpha]_D^{23}$ = -7.5 (c =1.0 in methanol)

^{1}H-NMR in $CDCl_3$:MeOD, 1:1, v/v

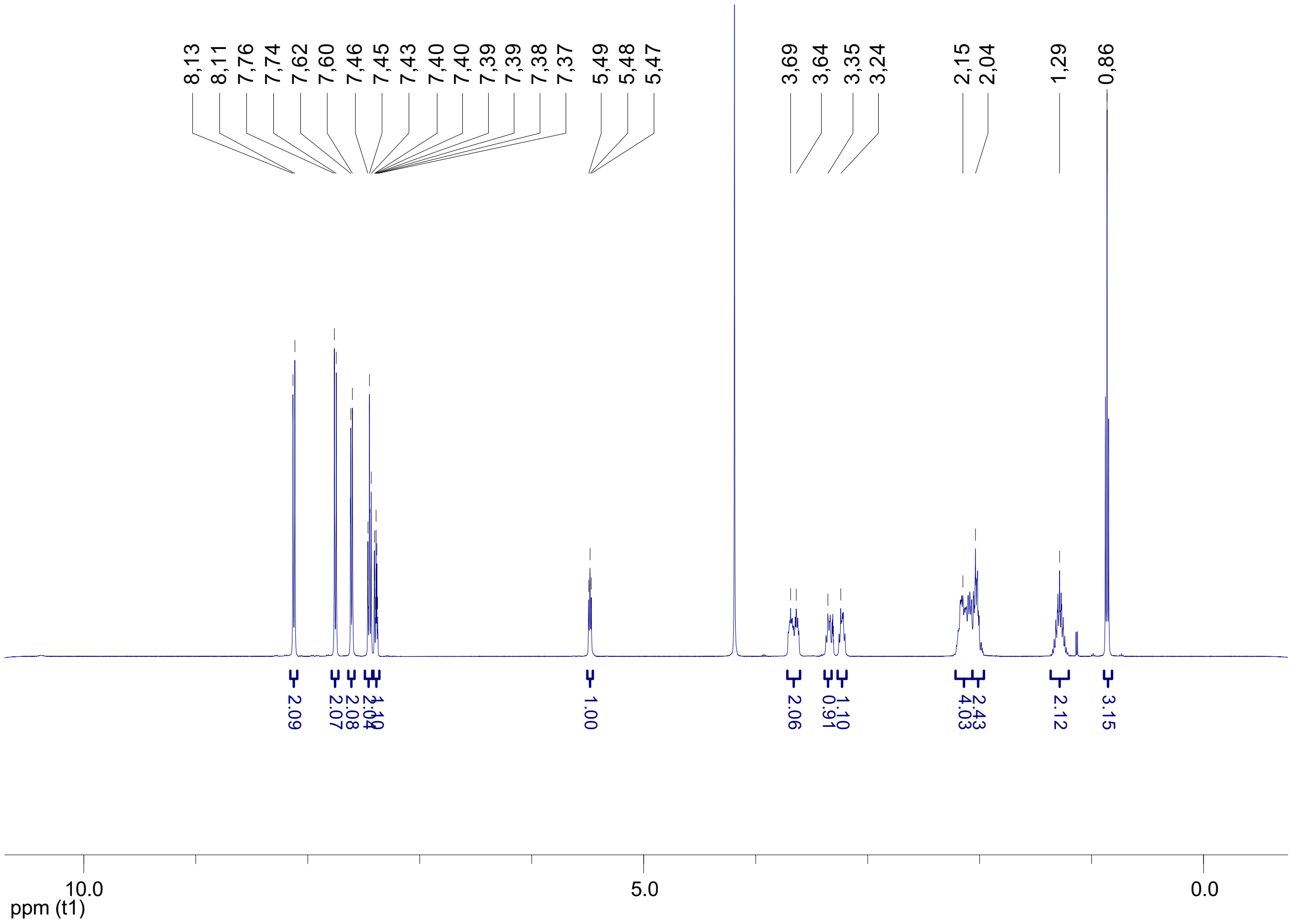

^{1}H-NMR in $CDCl_3$

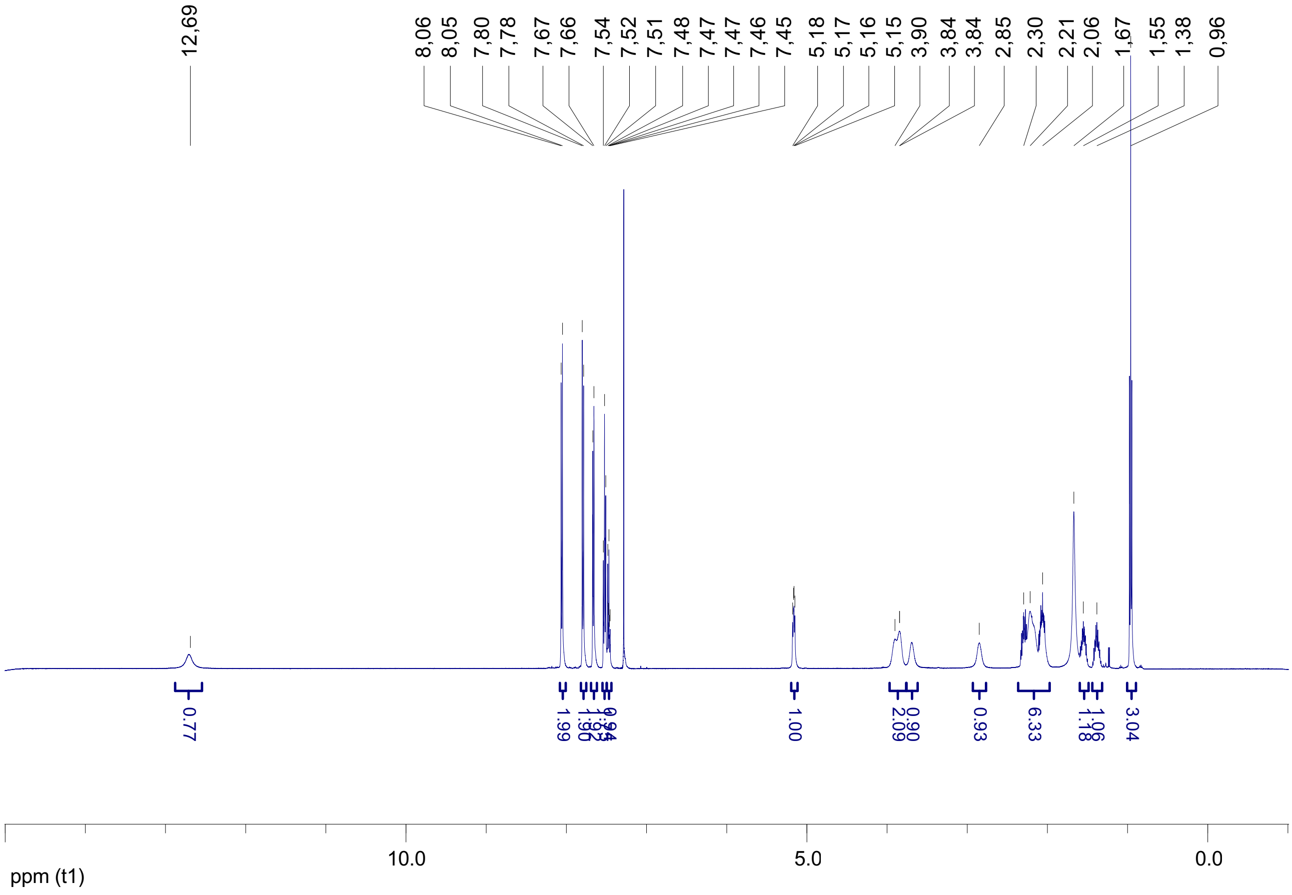

^{13}C-NMR in $CDCl_3$:MeOD, 1:1, v/v

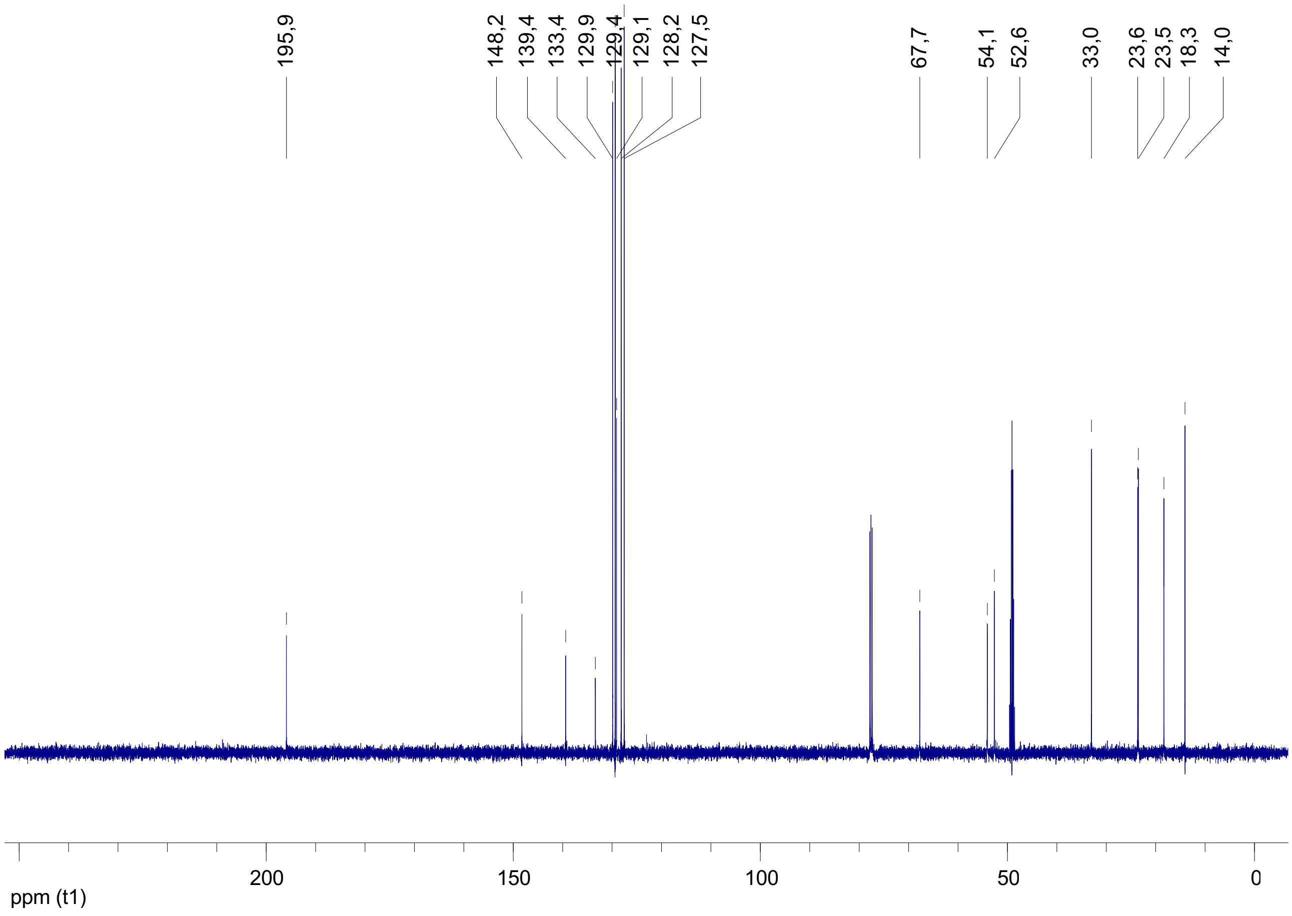

^{13}C-NMR in $CDCl_3$

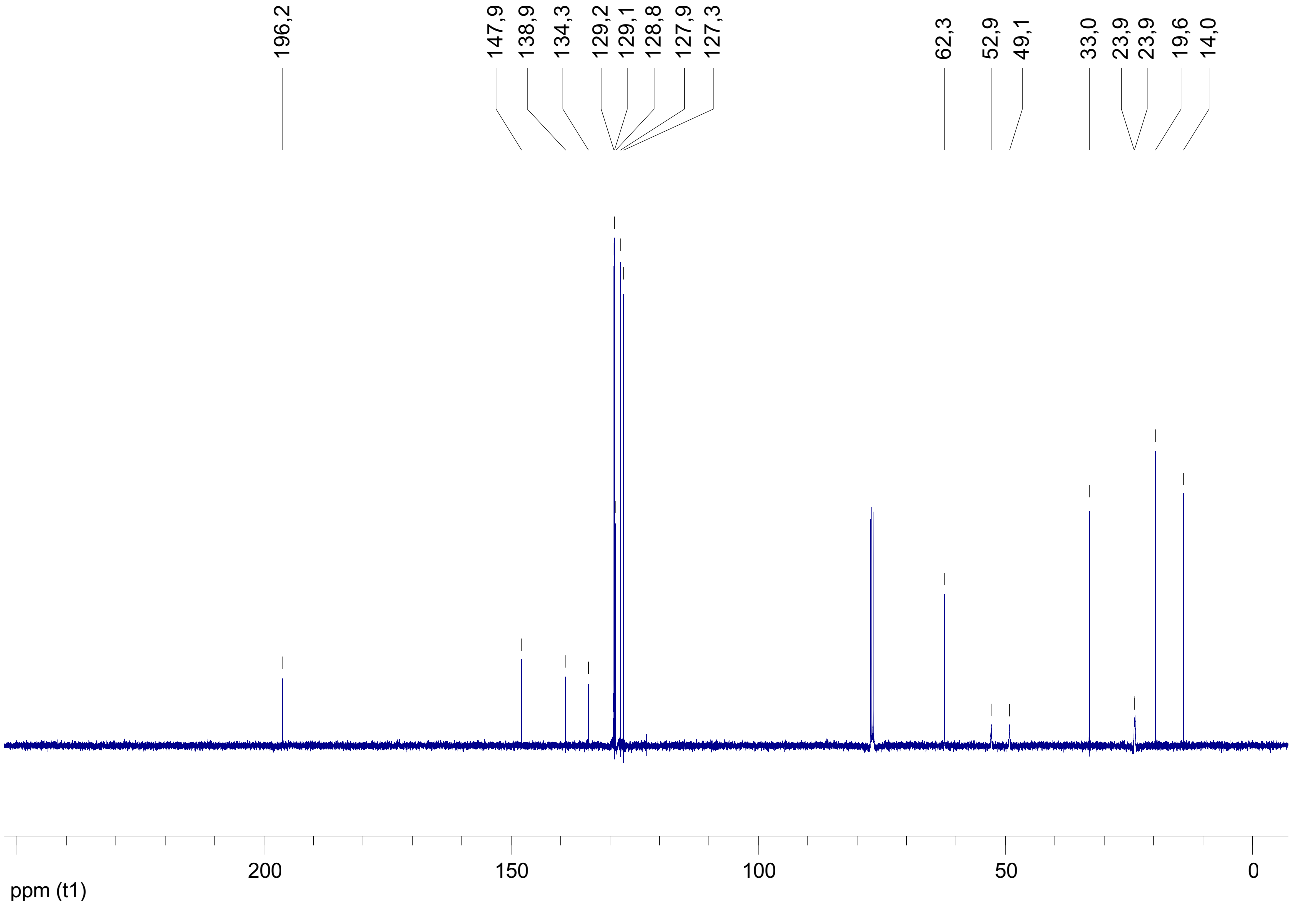

COSY

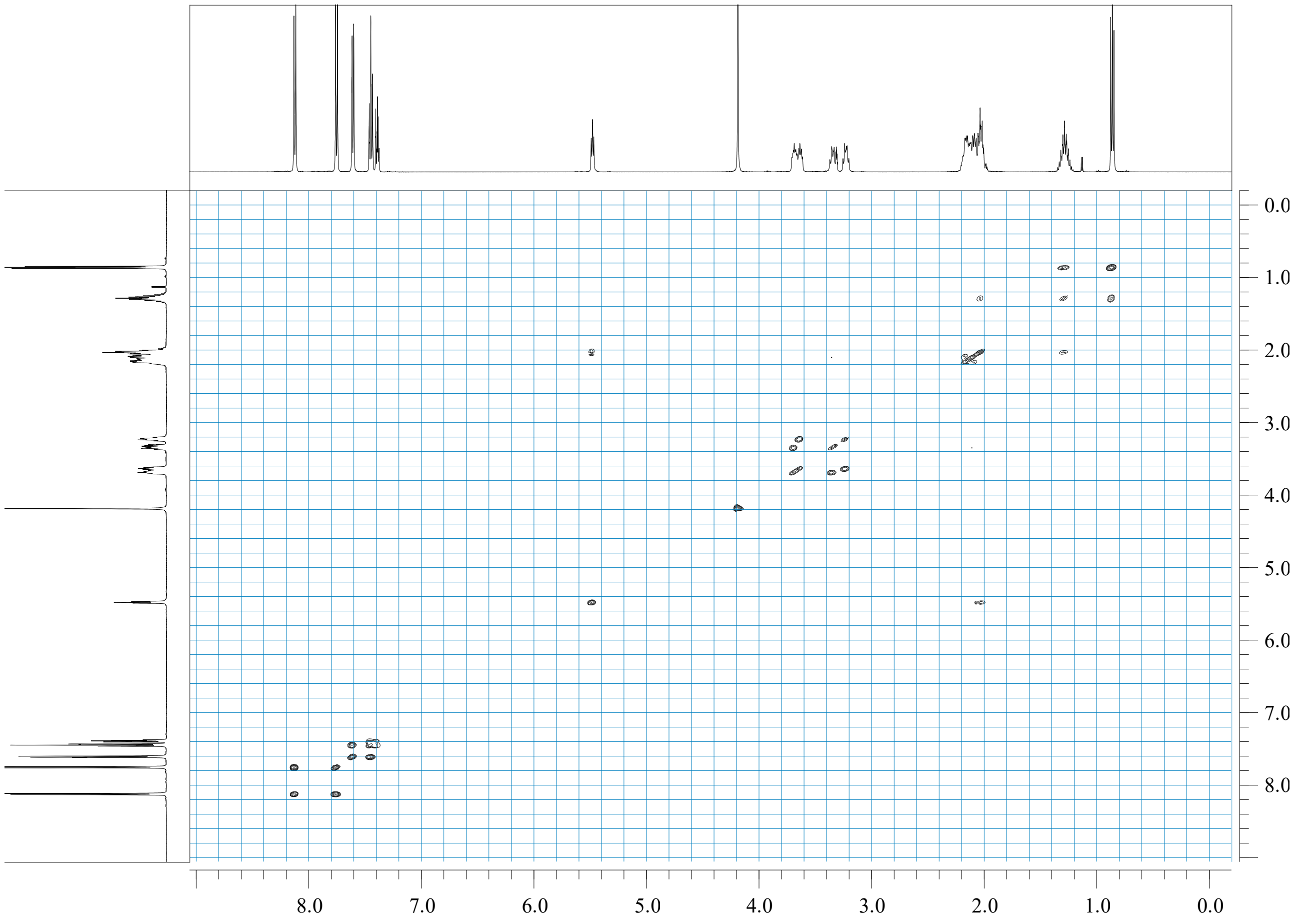

HSQC

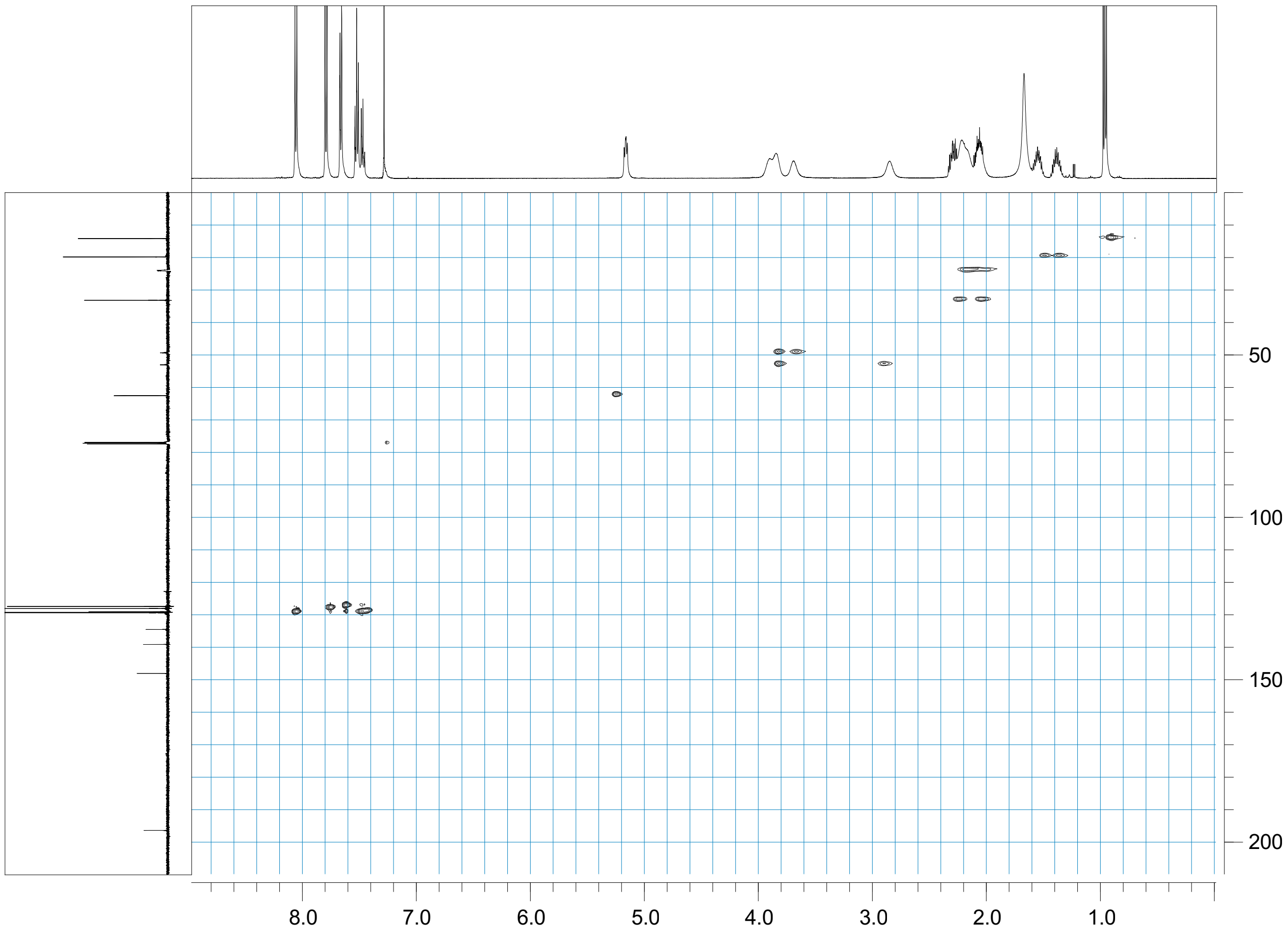

HMBC

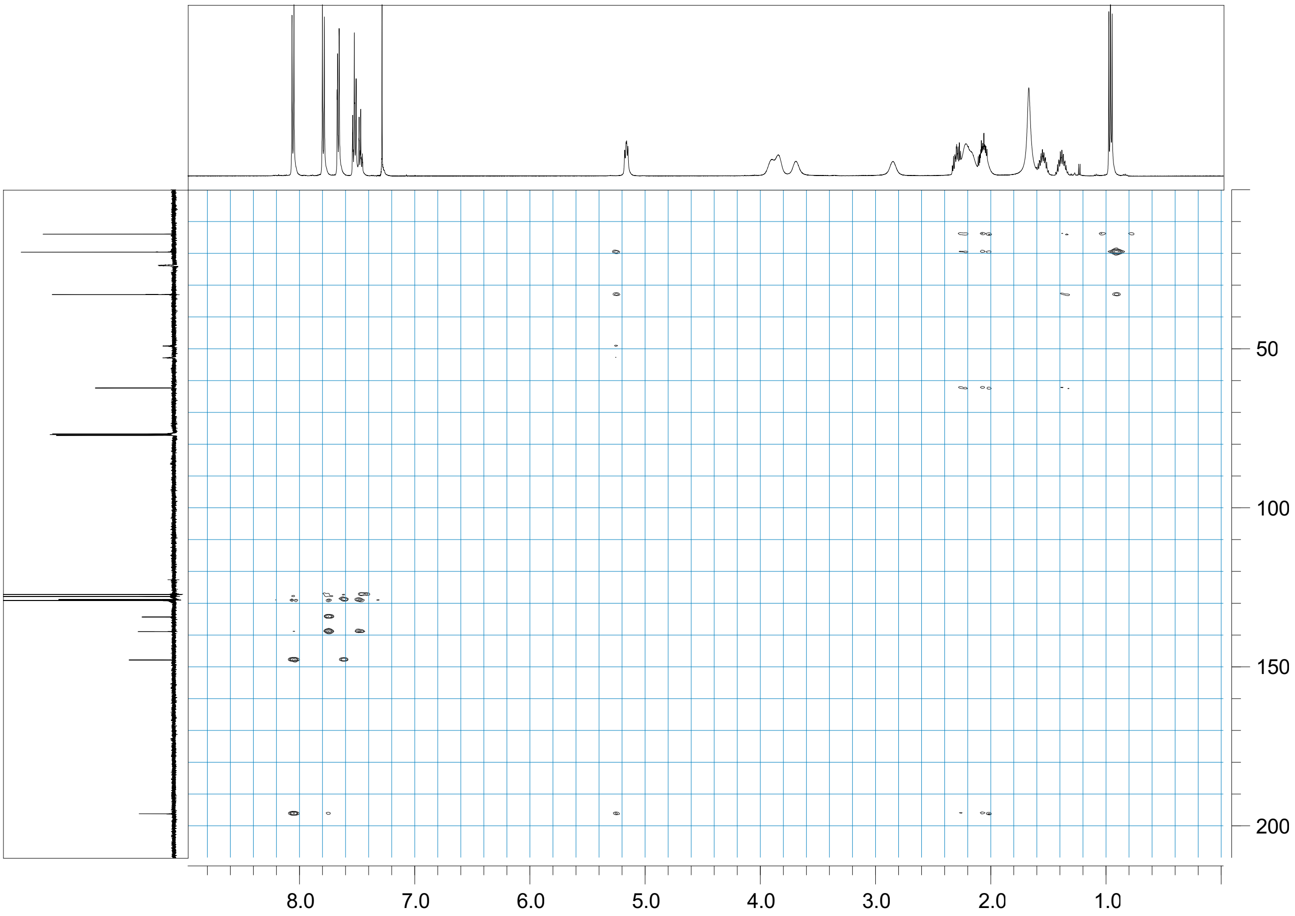

^{35}Cl-NMR in $CD_3OD:D_2O$

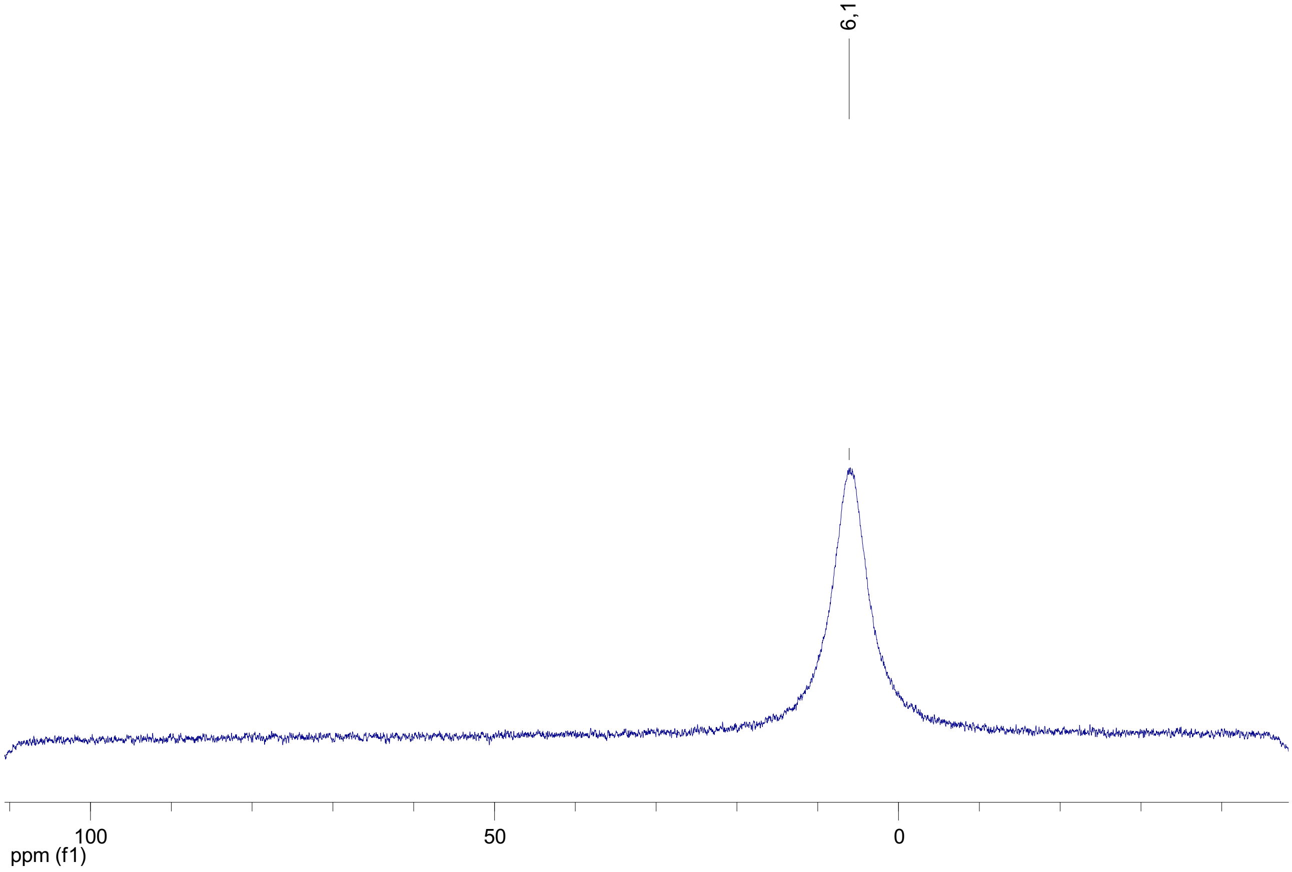

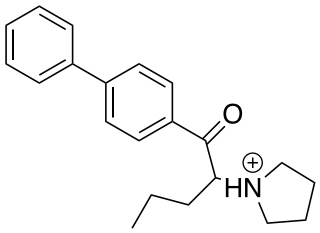

Chemical Formula: $C_{21}H_{26}NO^{+}$
Exact Mass: 308,2009
m/z: 308.2009 (100.0%), 309.2042 (22.7%), 310.2076 (2.5%)

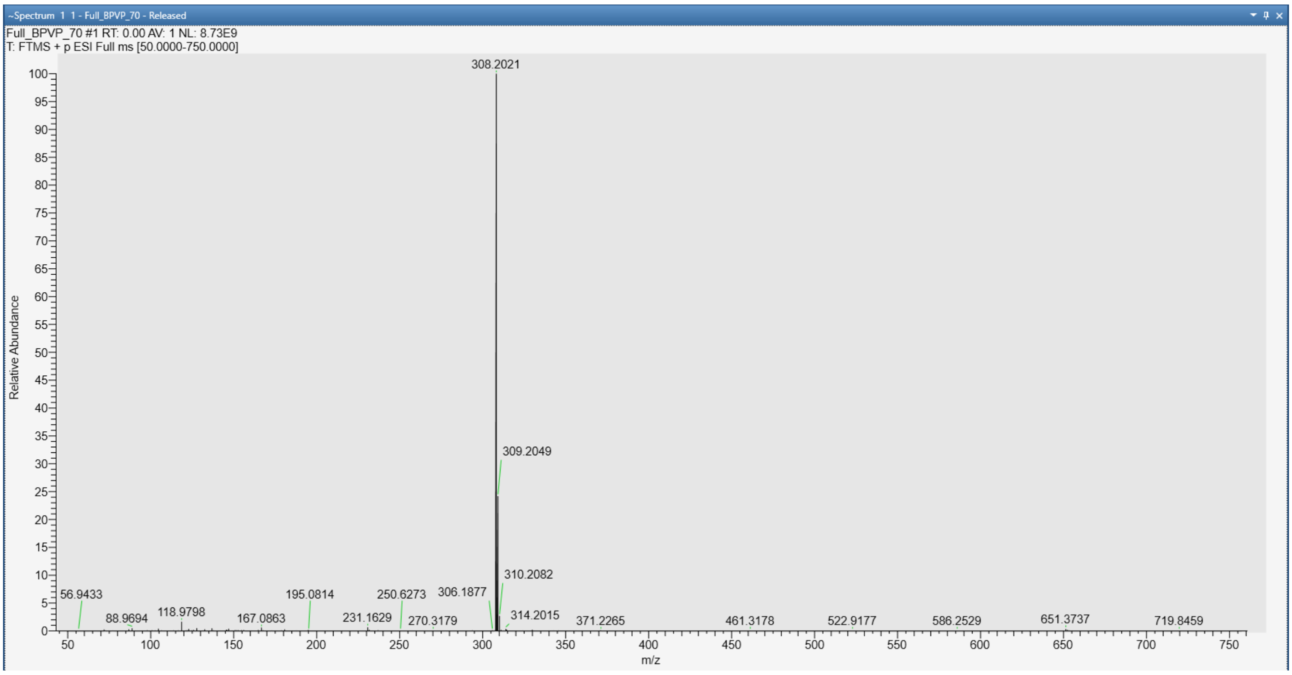

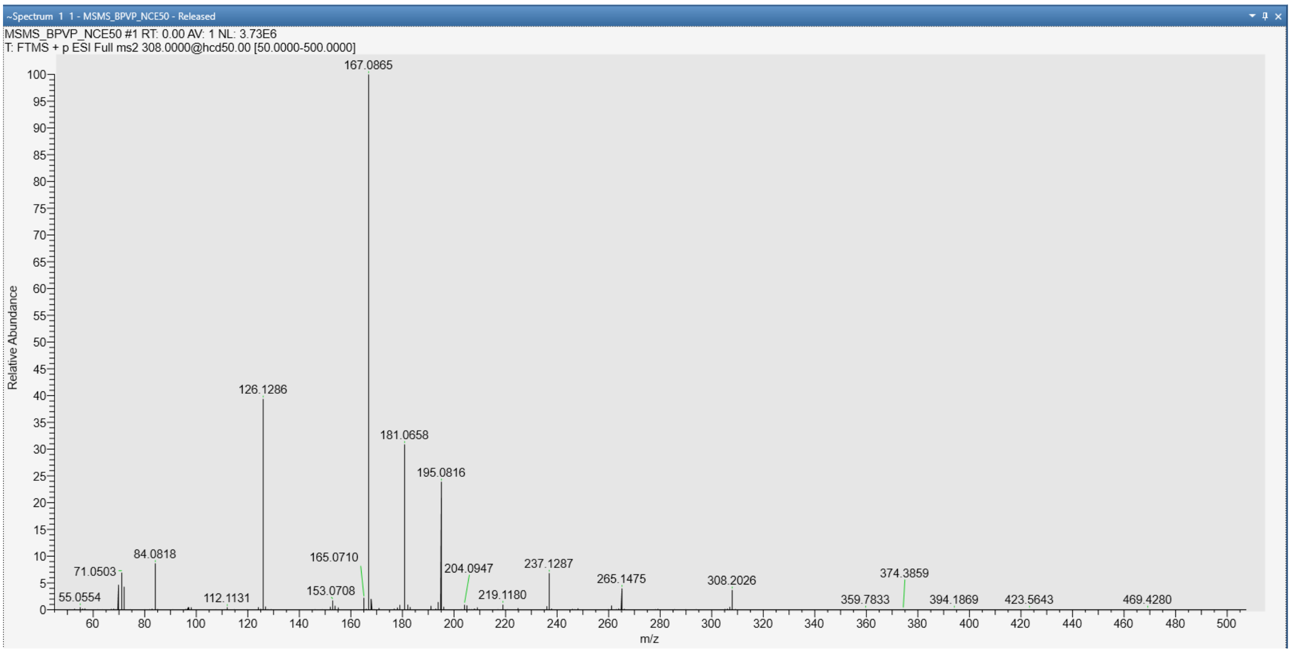

HRMS MS/MS of precursor ion

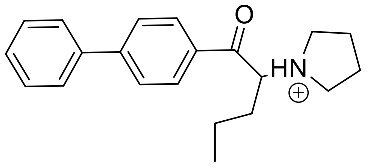

Chemical Formula: $C_{21}H_{26}NO^{+}$
Exact Mass: 308,2009

Precursor ion

Proposed fragments/ Suggested ion formula/ Calculated *m/z*	Measured *m/z*
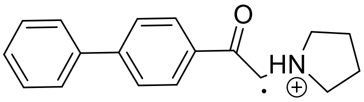 Chemical Formula: $C_{18}H_{19}NO^{\bullet+}$ Exact Mass: 265,1461	265.1475
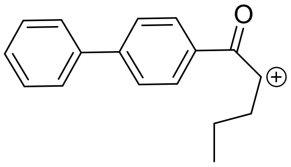 Chemical Formula: $C_{17}H_{17}O^{+}$ Exact Mass: 237,1274	237.1287
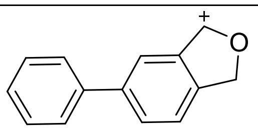 Chemical Formula: $C_{14}H_{11}O^{+}$ Exact Mass: 195,0804	195.0816
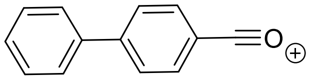 Chemical Formula: $C_{13}H_{9}O^{+}$ Exact Mass: 181,0648	181.0658
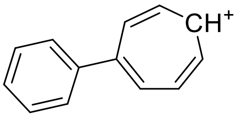 Chemical Formula: $C_{13}H_{11}^{+}$ Exact Mass: 167,0855	167.0865

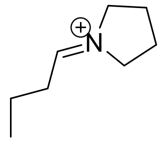 Chemical Formula: $C_8H_{16}N^+$ Exact Mass: 126,1277	126.1286
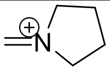 Chemical Formula: $C_5H_{10}N^+$ Exact Mass: 84,0808	84.0818

GC-MS

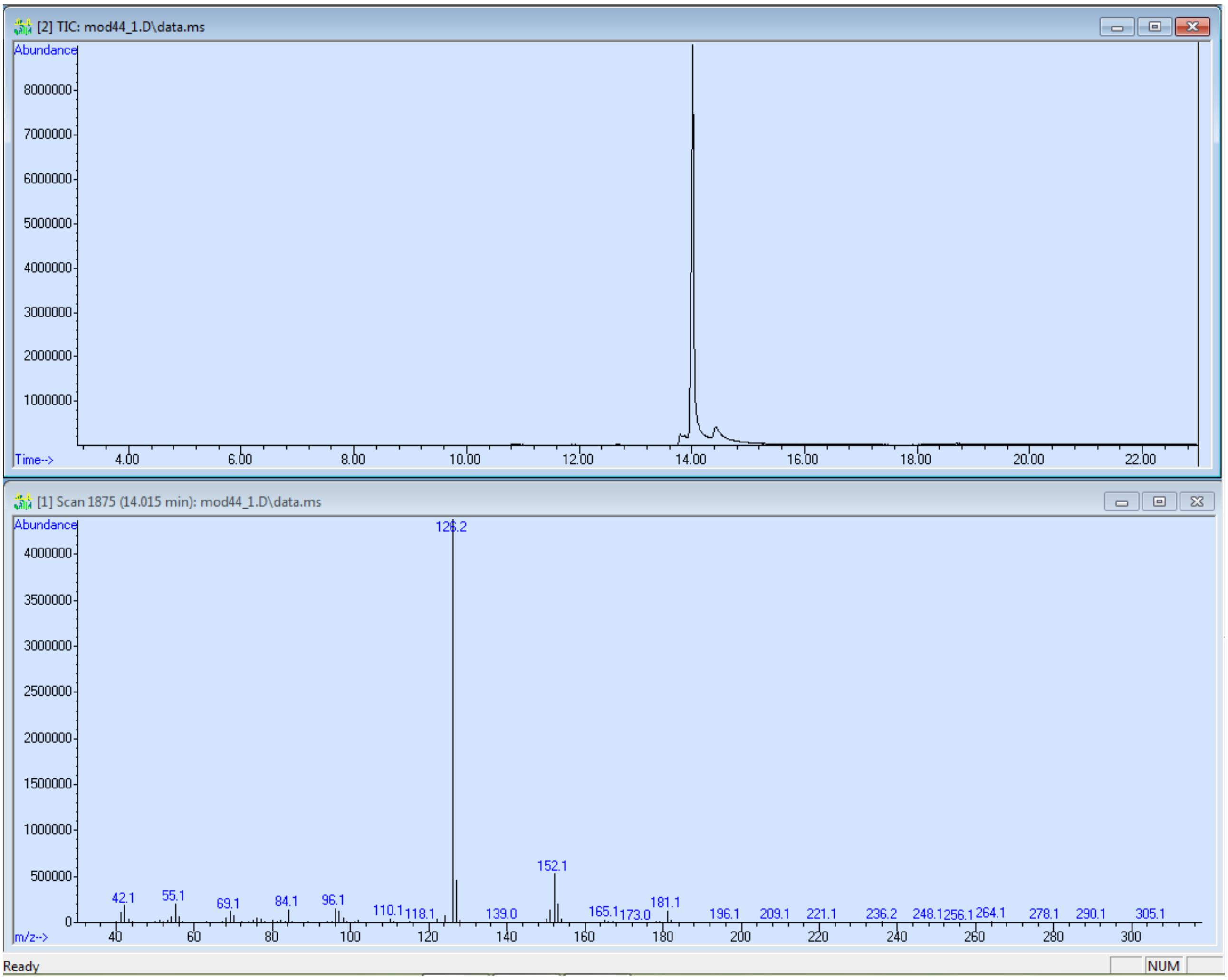
[2] TIC: mod44_1.D\data.ms
Abundance
8000000
7000000
6000000
5000000
4000000
3000000
2000000
1000000
Time-->
4.00
6.00
8.00
10.00
12.00
14.00
16.00
18.00
20.00
22.00
[1] Scan 1875 (14.015 min): mod44_1.D\data.ms
Abundance
4000000
3500000
3000000
2500000
2000000
1500000
1000000
500000
0
m/z-->
126.2
152.1
42.1
55.1
69.1
84.1
96.1
110.1
118.1
139.0
165.1
173.0
181.1
196.1
209.1
221.1
236.2
248.1
256.1
264.1
278.1
290.1
305.1
40
60
80
100
120
140
160
180
200
220
240
260
280
300
Ready
NUM
